# Extended Robotic Pulmonary Resections

**DOI:** 10.3389/fsurg.2021.597416

**Published:** 2021-02-22

**Authors:** Joshua A. Scheinerman, Jeffrey Jiang, Stephanie H. Chang, Travis C. Geraci, Robert J. Cerfolio

**Affiliations:** Department of Cardiothoracic Surgery, New York University Langone Health, New York, NY, United States

**Keywords:** robotic, lobectomy, non-small cell lung cancer, sleeve resection, pneumonectomy, neoadjuvant (chemo)radiotherapy

## Abstract

While lung cancer remains the most common cause of cancer-related mortality in the United States, surgery for curative intent continues to be a mainstay of therapy. The robotic platform for pulmonary resection for non-small cell lung cancer (NSCLC) has been utilized for more than a decade now. With respect to more localized resections, such as wedge resection or lobectomy, considerable data exist demonstrating shorter length of stay, decreased postoperative pain, improved lymph node dissection, and overall lower complication rate. There are a multitude of technical advantages the robotic approach offers, such as improved optics, natural movement of the operator's hands to control the instruments, and precise identification of tissue planes leading to a more ergonomic and safe dissection. Due to the advantages, the scope of robotic resections is expanding. In this review, we will look at the existing data on extended robotic pulmonary resections, specifically post-induction therapy resection, sleeve lobectomy, and pneumonectomy. Additionally, this review will examine the indications for these more complex resections, as well as review the data and outcomes from other institutions' experience with performing them. Lastly, we will share the strategy and outlook of our own institution with respect to these three types of extended pulmonary resections. Though some controversy remains regarding the use and safety of robotic surgery in these complex pulmonary resections, we hope to shed some light on the existing evidence and evaluate the efficacy and safety for patients with NSCLC.

## Introduction

With nearly a quarter-million lung cancers diagnosed in 2017 in the US, more than 80% of which were non-small cell lung cancer (NSCLC) (1), there is a substantial need for surgical resection for management of disease. In parallel with the growing need for pulmonary resection, there have been significant advances in thoracic minimally invasive surgery (MIS), with nearly 57,000 MIS lobectomies performed from 2002 to 2018, most of which in the latter decade (2). Furthermore, the rate of both video-assisted thoracoscopic surgery (VATS) and robotic thoracic surgery has increased significantly over the last decade. As the field of robotic thoracic surgery continues to develop, the breadth of surgery safely achievable using a robotic platform continues to expand. Complex surgeries with increased adhesions with associated increased risk for bleeding, such as post-induction pulmonary resections, are being performed robotically with more frequency. Other technically complex resections, such as sleeve resection or pneumonectomy, can also be safely completed via a robotic platform. There are numerous tangible advantages to the robotic platform, such as lower overall complication rate, less blood loss, decreased postoperative pain, shorter length of stay, as well as optimized pathologic staging due to a more complete lymph node dissection in comparison to thoracotomy (3). In a large multi-center study with 1,336 robotic lobectomies performed, Cerfolio et al. demonstrates median blood loss of 50 ml, mean operative time of 136 min, and median length of stay of 3 days (4), all of which have improved over the past 2 years (publication pending). Given the benefits of robotic surgery in early-stage NSCLC resection, there is likely a similar benefit of the robotic approach for these extended resections.

## Post-Induction Robotic Pulmonary Resections

### Indications for Neoadjuvant Therapy

The ideal treatment for early-stage NSCLC is complete surgical resection. However, the management increases in complexity for advanced-stage disease. For non-superior sulcus lung cancer with no mediastinal lymph node disease and resectable disease, the standard treatment is surgery, with possible adjuvant systemic therapy and/or radiation depending on the final pathologic stage. Assuming negative pathologic margins, current NCCN guidelines for NSCLC state adjuvant chemo is appropriate for stages IB (high-risk patients), IIA, and IIB. However, for patients with N2 (mediastinal disease), neoadjuvant treatment with chemotherapy or chemoradiation therapy followed by restaging and surgical resection is the standard treatment (5). In 1992, the National Cancer Institute performed a study aimed at evaluating the effect of neoadjuvant chemotherapy on NSCLC. Pass et al. randomized Stage IIIA (N2) disease patients to surgery followed by radiotherapy or neoadjuvant cisplatin/etoposide followed by surgery. Of the 27 patients followed, there was a trend toward improved survival in the neoadjuvant arm, 28.7 months with neoadjuvant vs. 15.6 months. The recurrence rate and disease-free survival also favored the neoadjuvant arm (6). These data are reinforced further by a 2010 *Journal of Thoracic Oncology* meta-analysis that looked at 13 randomized control trials treating NSCLC patients with neoadjuvant + surgery vs. surgery alone. The data illustrated that neoadjuvant benefited stage III NSCLC patients significantly with respect to comparative survival, with this subgroup analysis of more than 1,500 patients revealing a combined HR = 0.84, *p* = 0.005 (7) (Table 1).

**Table 1:** Role of neoadjuvant chemotherapy for advanced NSCLC.

**Trial**	**Study type**	**Date**	***n***	**Findings**
Pass et al. (6)	Single-center-RCT	1987–1992	*N* = 13, neoadj. chemo+ surgery, 14, surgery + RT	0% operative mortality both groups 28.7 month survival vs. 15.6 month survival (Neoadj. vs. surgery), trend toward survival in neoadj 12.7 vs. 5.8 months DFS (Neoadj vs. surgery), trend favoring neoadj 11/12 surgery + RT recurrence vs. 8/11 neoadj recurrence Complete tumor and nodal resection rates similar
Song et al. (7)	Meta-analysis, retro	1985–2004	*N* = 1,637 neoadj.+ surgery, 1,587 surgery alone	Survival improved in neoadj. Arm, HR = 0.84, *p* < 0.005, in all-comers and Stage III NSCLC pts.
Rosell et al. (8) Roberts et al. (9)	Prosp. RCT, Retro. cohort	1989–1991 1997–1999	*N* = 30 neoadj. + surgery + RT, 30 surgery alone + RT *N* = 34 neoadj., 67 surgery alone	26 vs. 8 months, neoadj. vs. surgery alone, median survival20 months vs. 5 months, neoadj vs. surgery alone, DFSRecurrence rate 56 vs. 74%, neoadj. vs. surgery aloneIncreased incidence in life-threatening complications post-op in neoadj. group, major complications 47.1 vs. 19.1%, Increase in tracheostomy rate in neoadj. group2 deaths in neo adj. group within 90 days, 0 in surgery-alone group

### Surgical Resection After Neoadjuvant Therapy

Induction therapy for NSCLC poses unique technical challenges for the minimally invasive thoracic surgeon. Dense adhesions and fibrosis in the setting bulky tumors with inflamed lymphadenopathy can create difficult and hazardous dissections. While the initial report in 1994 evaluating the efficacy of induction chemotherapy in stage IIIA disease showed no difference in postoperative mortality between the neoadjuvant vs. upfront surgery arm, there was a high 30-day mortality of 7% in each arm (8). However, there are some data demonstrating increased postoperative 90-day mortality and overall complication rate. Roberts et al. demonstrated increased life-threatening complications (26.5 vs. 6.0%), major complications (47.1 vs. 19.4%), and 90-day mortality (5.9 vs. 0%) in patients who had neoadjuvant treatment vs. upfront surgery, respectively (9).

Some data exist comparing minimally invasive to open resection for NSCLC after induction therapy. Fang et al. demonstrated no significant difference in postoperative morbidity or overall survival in the VATS vs. thoracotomy group, though VATS was associated with decreased postoperative pain with the compromise of decreased number of lymph nodes (10). Park et al. retrospectively reviewed 428 patients with Stage II-IIIA NSCLC after they underwent lobectomy following neoadjuvant chemo/chemoradiotherapy. This group was divided into two cohorts, resection via conventional thoracotomy (*N* = 397) vs. MIS technique (*N* = 31, 17 robotic, 14 VATS) (11). Three-year overall and disease-free survival were comparable between two groups, as was the complication rate. Length of stay was shorter for the MIS group. R0 resection rate was similar between both groups, 94% for thoracotomy and 97% for MIS. Conversion to thoracotomy occurred in 8/31 (26%) MIS cases due to extent of disease, severe adhesions, and bleeding (11).

Data looking at MIS resections for neoadjuvant NSCLC have also shown similar metrics regarding a high conversion rate to open resection. Huang et al. evaluated 43 patients with stage IIA-IIIB NSCLC with neoadjuvant treatment who underwent VATS pulmonary resection. Most resections were a lobectomy, though there were nine bronchial sleeve lobectomies, five bi-lobectomies, and four pneumonectomies performed. Survival for the entire cohort at 1, 2, and 3 years were 94, 79, and 65%, respectively. Forty patients received an R0 resection. There was a 2.3% mortality (1/43 patients), and 5 patients had postoperative complications. While no patient was converted to “conventional thoracotomy,” a “hybrid VATS” was performed in 7 (16.7%) of the cases, due to proximity of the tumor to large vessels and/or bronchi, in the setting of mod-severe inflammation and fibrosis due to the neoadjuvant therapy (12). A multi-center retrospective review by Cerfolio et al. evaluated 223 patients with occult or evident N2 disease undergoing robotic-assisted resection. Of these, 15.2% received neoadjuvant therapy, and 8 patients in that group also received pre-operative radiotherapy. Major complications (10.1%) were similar between the neoadjuvant group and surgery-upfront group. R0 resection rate was 98.1%, with a 0% 30 and 90-day mortality in the neoadjuvant group. Of the neoadjuvant resections, 15% converted to thoracotomy. No conversions were for bleeding, but generally for oncologic resection due to discovery of extensive mediastinal lymph node involvement (13) (Table 2).

**Table 2:** Outcomes for minimally invasive vs. open resection after neoadjuvant therapy for NSCLC.

**Trial**	**Study type**	**Date**	***n***	**Findings**
Fang et al. (10)	Retro, SC cohort	2013–2017	*N* = 67 open, 14 VATS	Less post-op pain, decreased absolute LN harvest in VATS group OS and DFS similar, 32.7 (open) vs. 31.8 (VATS) months for DFS
Park et al. (11)	Retro, SC cohort	2002–2013	*N* = 397 open, 31 MIS (17 robotic,14 VATS)	R0 resection rate and post-op morbidity similar LOS shorter in MIS group (4 vs. 5 days) DFS and OS similar, 49 vs. 42% DFS, MIS vs. open 26% conversion-to-open rate in MIS group
Huang et al. (12) Bott et al. (14) Cerfolio et al. (15)	Retro, SC cohort MC, single-arm cohort Retro, MC	2006–2012 2015–2016 2007–2016	*N* = 43 *N* = 20 *N* = 223, 34 received neoadj.	28 lobectomy, 9 sleeve, 5 bi-lobectomy, 4 pneumonectomy 16.7% conversion rate to “Hybrid-VATS,” remainder VATS Median blood loss 253 cc, op. time 160 min, hosp. stay mean 5.45 days 11.9% incidence post-op complications 1 perioperative death, 3-year survival 65% 15 lobectomy, 2 pneumonectomy, 1 sleeve, 1 bi-lobe 13 attempted VATS/robotic, 54% conversion-to-open Median op. time 228 min, LOS 4 days, no periop mortality, 50% post op morbidity rate All 34 had R0 resection, 15% conversion rate (not due to bleeding), 12% major post-op complications 3-year survival 60.1% in neoadj. group OS much better in pts with <2 + LNs

Surgery after induction immunotherapy also poses similar risks. Broderick et al. evaluated outcomes of 20 stage I-IIIA NSCLC patients who received with neoadjuvant Nivolumab followed by pulmonary resection, 13 of which were initially attempted via VATS or robotic. There was 0% perioperative mortality, and 50% morbidity, most commonly atrial fibrillation. Three-quarters of the patients underwent lobectomy, while another 10% received a pneumonectomy. Of the 13 procedures attempted via MIS, seven procedures (54%) converted to thoracotomy. Some of the conversions were due to hemorrhage and/or dense adhesions in the chest. The remainder of conversions were due to severely inflamed mediastinal and hilar nodal stations (14).

Current data demonstrate that induction treatment, with chemotherapy, radiation, or immunotherapy, can lead to increased need to convert from minimally invasive to open resection. However, these studies also demonstrate that minimally invasive pulmonary resection post-induction therapy has similar overall survival compared to open resection. Thus, in the properly chosen patient with locally advanced disease, robotic resection post-induction is safe, effective, and comparable in survival and complications when compared to conventional open resection.

### Robotic Techniques for Surgery After Neoadjuvant Treatment

Robotic pulmonary resections at our institution are performed on Da Vinci Xi robot surgical system (Intuitive Surgical Inc., Mountain View, CA). Bronchoscopy is performed to ensure correct placement of the double-lumen tube, and the patient is placed in lateral decubitus position. We employ a standardized set up: a four-arm approach and a 0-degree scope, with congruent port placement in the 8th intercostal space, with one assist port for the bedside assistant that is also utilized as air seal where CO_2_ is insufflated into the pleural cavity (16). In general, the anterior-most port (a 12-mm port) is placed one rib higher than the other three robotic ports to avoid injury or stunning the nerve to the upper rectus abdominis muscle. A multi-level intercostal nerve block is performed prior to docking.

Dissection usually begins with careful exploration of the chest cavity to ensure there are no pleural metastases or unsuspected free fluid. The inferior pulmonary ligament is divided, with the lung retracted superiorly with the 4th arm, and station 9, and then the eight lymph nodes are harvested. Following this, the subcarinal lymph node packet is entirely removed, as are all of the stations 2–4 if on the right side, or 5–6 if in the left chest. In post-induction resections, we pay close attention to evaluating which aspect of the dissection is going to be most challenging due to adhesions. Location of the mass, lymphadenopathy, and areas of prior positive nodal disease are evaluated with relation to the hilar vessels and bronchus. We prefer to start with a posterior dissection. On the left side, the posterior view allows for identification of the PA, as well as the A2 and A6 PA branches to be identified to help completely open the posterior fissure. On the right side, the posterior approach allows for the right upper lobe bronchus to be encircled and divide prior to any PA branches being ligated. These tricks provide safer PA dissection. Prior to dissection of these area, hilar control can obtained for select cases, with careful dissection around the pulmonary veins using blunt and bipolar energy, as well as dissection of the proximal pulmonary artery. If the tumor is quite proximal, an intra-pericardial technique is used to encircle the pulmonary artery and the pulmonary veins. After this control is obtained, the dissection is done. If significant bleeding is encountered from the pulmonary artery or vein, the operator can effectively clamp the hilum. During dissection around of vascular structures, multiple sponges are in the chest, ready to hold pressure if needed. A thoracotomy tray should be in the room, and blood products should be on hold.

Our group has written about the approach and mindset of the robotic thoracic surgeon before with respect to conversion to open for massive bleeding. The paradigm of preparation, pressure, patience, poise, products, partner, and prolene applies here as well, with the addition of already having had hilar control (15). We find these to be the salient points of performing robotic post-induction pulmonary resection, with the most vital being the potential for massive large vessel hemorrhage and the need for prophylactic hilar control beforehand.

## Robotic Sleeve Resection

### Indications for Sleeve Resection

Centrally located NSCLC presents a unique challenge for obtaining an R0 resection. Airway involvement including the proximal lobar bronchus or the main stem airway precludes the ability to perform a lesser anatomic resection and has historically necessitated pneumonectomy. Sleeve resection of the airway was developed as an alternative to pneumonectomy. This was traditionally only offered to frail patients with poor pulmonary reserve for fear of a poor oncologic resection and close margins. Recently, however, sleeve resection has become more accepted as the primary surgical option, even in those who can tolerate pneumonectomy (16). Currently sleeve resection is indicated for any stage I and II centrally located tumors where sleeve reconstruction is technically feasible (17, 18).

The benefits of sleeve resection for early-stage central tumors is highlighted in a large single institution retrospective series conducted in France, which evaluated 1,230 patients with 184 sleeve resections. The authors demonstrate significantly improved 5-year survival (52 vs. 31%) as well as decreased locoregional recurrence (22 vs. 35%) in stage I/II patients of sleeve resection compared to pneumonectomy (19). Okada et al. similarly reported better for 120 matched patients, 60 sleeves vs. 60 pneumonectomies, with a 48 vs. 28% 5-year survival (20). National data from France comparing 941 sleeve lobectomies to 5,318 pneumonectomies resulted in the recommendation for sleeve lobectomy whenever feasible in early-stage cancer based on equitable long-term mortality and possibly better results when propensity matched (18) (Table 3).

**Table 3:** Comparison of sleeve lobectomy and pneumonectomy outcomes.

**Trial**	**Study type**	**Date**	***n***	**Findings**
Deslauriers et al. (19)	Single-center cohort	1972–2000	184 SL 1046 PN	1.3 (SL) vs. 5.3% (PN) Op mortality 52 (SL) vs. 31% (PN) 5Y survival 63 (SL) vs. 43% (PN) 5Y survival in Stage I/II NO survival difference for stage III 22% (SL) vs. 35% (PN) Locoregional recurrence
Okada et al. (20)	Single-center cohort	1984–1998	151 SL 60 SL matched 60 PN	0 (SL) vs. 2% (PN) Op mortality 13 (SL) vs. 22% (PN) morbidity 8 (SL) vs. 10% (PN) recurrence 48 (SL) vs. 28% (PN) 5Y survival
Pages et al. (18)	Nationwide cohort	2005–2014	941 SL 5318 PN	5 (SL) vs. 5.89% (PN) Op mortality 1.63 HR long-term survival PN vs. SL (matched data, *p* = 0.002) 71.8 (SL) vs. 60.76% (PN) 3Y DFS

Patients with lesions involving the proximal portion of the pulmonary artery warrant special consideration and have been shown to benefit from pulmonary arterioplasty with or without bronchial sleeve resection. Defects in the artery can vary and reconstruction has been described with patch reconstruction, primary anastomosis, or interposition graft (21). Rendina et al. have reported a 20-year experience with pulmonary arterioplasty during lobectomy in 105 patients. Their series included 47 pulmonary artery sleeves and 65 PA reconstructions associated with bronchial sleeve. Operative mortality was 0.95% and 5-year survival was 44%. Prior concerns of PA thrombosis or hemorrhage were rare with a single case of thrombosis. Long-term disease-free survival was equivalent to that expected of lesser resections stage for stage (22, 23). Meta-analysis of sleeve lobectomy with pulmonary arterioplasty has also demonstrated equivalent operative risk, complication rate, and long-term mortality to isolated sleeve lobectomy and trend toward improved outcomes compared to pneumonectomy (24).

### Minimally Invasive Sleeve Resections

The benefits of minimally invasive surgery have been well-documented (25). As reiterated above, the advent of robotic-assisted surgery has continued to improve complication rates, length of stay, and 30-day mortality when compared to video-assisted surgery (26). Sleeve resection had previously been limited to an open approach, barring a few specialized centers, given VATS's limited maneuverability and lack of depth perception. Robotic-assisted bronchoplasty was first described in cadaver models before being applied in a hybrid VATS/RATS fashion (27). The challenges of bronchoplasty are well-suited to the robotic platform and allow for precise intrathoracic suturing.

Application robotic systems have occurred over the past decade. Few institutions have published series reviewing their results. Jiao et al. have reported the largest series with 67 patients with broad indications for sleeve lobectomy, including those with peri-bronchial lymph node metastases. They demonstrate safety and feasibility of the procedure, with no 90-day mortalities, few complications, and no conversions to open. Major complications included one anastomotic stricture, one chylothorax, one stroke, and one patient readmitted for pneumonia (28). Our group has reported the second largest series of 23 patients with no short-term mortality and minimal morbidity, consisting of one conversion to open for concern of anastomotic tension, one readmission for pneumonia, and one transfusion for asymptomatic anemia. Over a median follow-up of 18 months, no patients had locoregional recurrence (29). Pan et al. had previously reported a similar series of 21 robotic sleeve resections with similar results, although with one 30-day mortality and marginally higher rates of complications (30). Recent retrospective review of robotic sleeve compared to VATS and open show at least parity of short-term outcomes in weighted matched patients and suggest decreased bleeding, length of stay, and operative time (31) (Table 4). While short-term oncologic results seem promising, long-term data remains to be seen.

**Table 4:** Outcomes from robotic sleeve resection.

**Trial**	**Study type**	**Date**	***n***	**Findings**
Jiao et al. (28)	Series	2014–2018	67	0% 90-day mortality 0% conversion to open 13% complication (1 bronchial stenosis, 2 prolonged air leak, 1 pneumonia, 1 chylothorax, 1 CVA) Mean OR time 166.5 min 6.8 days average LOS
Geraci et al. (16)	Series	2010–2019	23	0% 90-day mortality 100% R0 resection 1 conversion to open for anastomotic tension 9% morbidity (1 pneumonia, 1 transfusion) 0% recurrence in median follow-up 18 months Median OR time 205 min 3 days median LOS
Pan et al. (30)	Series	2014–2015	21	4.8% 30-day mortality 90.5% R0 resection 4.8% conversion to open for calcified nodes 19% complication (2 pneumonia, 2 pyothorax, 1 anastomotic leak) Mean OR time 158 min 10.7 days average LOS
Qiu et al. (32)	Retro-Cohort	2012–2017	188 SL 49 robotic 73 VATS 66 open	119 (R) vs. 182 (VATS) vs. 222 (open) EBL 200 (R) vs. 291 (VATS) vs. 240 (open) OR time 7.7 (R) vs. 10.2 (VATS) vs. 10.2 (open) LOS 4.2 (R) vs. 7.2 (VATS) vs. 6.9 (open) Tube drainage days 100% (R) vs. 91.2 (VATS) vs. 98.3 (open) Negative margins No difference in mortality or morbidity before and after propensity matching

### Robotic Technique for Sleeve Resection

Our approach for robotic sleeve lobectomy consists of a completely portal robotic approach with the Da Vinci XI system (Intuitive Surgical Inc, Mountain View, CA) and a 0° camera. Prior to port placement, bronchoscopy is done to map tumor extent and endobronchial involvement. Four robotic ports are placed and one assistant port are placed in standard position for a lobectomy, as described above.

A right upper lobectomy is described as it is the most common concomitant lobectomy performed. A mediastinal lymphadenectomy is performed, with careful attention to clearing the entire subcarinal lymph node packet, in order to visualize the takeoff of the right and left mainstem bronchi. The anatomic triangle around the right upper lobe bronchus takeoff is dissected clean by resecting the level 10 lymph node associated. The fissure is then dissected free with either bipolar if complete or tunneled and the parenchyma stapled to reveal the ongoing artery. The posterior ascending artery and azygos vein are stapled to allow for complete mobilization of the trachea and to prevent fistula to the bronchial anastomosis. The F_i_O_2_ is lowered to 21%, and then the RUL bronchus is transected with unipolar scissors. The remainder of the lobectomy is completed after division of the truncus anterior and the posterior fissure. Video of this procedure is available at https://www.youtube.com/watch?v=0_k-3Sgro5E.

For lesions that require pulmonary artery reconstruction, proximal, and distal control are obtained with vessel loops and flexible vascular clamp (Cygnet; Peters Surgical, Bobigny, France) inserted through a separate incision and contoured to the chest wall or a reliance Bulldog clamp (Scanlan, Saint Paul, MN) through the robotic ports. Distal control is obtained similarly or by clamping the pulmonary vein. Heparin was not routinely given unless clamp time was prolonged. Bronchotomy was done under infrared visualization and bronchoscopy (Firefly; Intuitive Surgical, Sunnyvale, CA). Proximal bronchotomy is done first to visualize the distal extent of the lesion. Frozen section of the proximal and distal bronchus is performed, and re-resection is done if margins are positive.

For the bronchial anastomosis, the airway is oriented so the membranous portion is parallel to the spine and two continuous running Stratafix sutures (Johnson & Johnson, New Brunswick, NJ) are used. The cartilaginous portion of the anastomosis is completed first from 6 to 12 o'clock on the proximal airway (junction to junction of the cartilaginous and membranous portions) (video available at https://www.youtube.com/watch?v=HVnBIeAgXdU) before the lung is retracted superiorly and anteriorly to run a second suture along the membranous portion. Pneumostasis is tested by reinflation under saline and, if an air leak is present, a single repair stitch is placed. Routine buttressing is not done unless the patient underwent neoadjuvant therapy (29).

## Robotic Pneumonectomy

### Indications for Pneumonectomy

Pneumonectomy remains an operation of last resort given the associated morbidity and mortality (29). Of the elective pulmonary resections, it entails the highest risk with operative mortality ranging from 3 to 12% with most recent guidelines stating a risk of 7% or less as acceptable (33). Mortality risk increases with operation for inflammatory conditions and prior induction therapy with rates reported of over 12% (34). Cardiovascular complications are also significantly higher than other lesser resection with rates reported of 20–60% with many life-threatening requiring immediate intervention (35). Other morbidities include pneumonia, bronchopleural fistula, recurrent laryngeal nerve palsy, pulmonary embolus, and prolonged extubation (36, 37). Centrally located NSCLC with involvement of the hilar structures from direct tumor invasion or extensive hilar nodal disease requires pneumonectomy for R0 resection. Other indications include multi-lobar disease or metachronous lesions in remaining lobes. Pneumonectomy also remains as backup when lung preservation is found to be an unacceptable oncologic resection in patients with adequate pulmonary reserve (29).

### Minimally Invasive Pneumonectomy

As previously stated, the benefits of MIS have been validated and include decreased blood loss, length of stay, postoperative pain, and equivalent oncologic outcomes. The expansion of VATS or robotic pneumonectomy, however, has been slow and hindered by technical, safety, and oncologic concerns. Thus, there have been few series of VATS pneumonectomies and only case reports of robotic-assisted operations.

Thoracoscopic pneumonectomy has been described in three prior series all with fairly similar results. They largely conclude VATS pneumonectomy as a feasible and safe procedure with equivalent oncologic outcomes, albeit with a high conversion rate. Battoo et al. reviewed 107 single-center patients (67 VATS) with similar rates of postoperative complications, no major intraoperative bleeding, a conversion rate of 16%, and decreased 1-year pain (38). Liu et al. matched 64 open cases to 32 VATS pneumonectomies with similar postoperative complication rates and longer OR times (39). The only multi-center review conducted by Yang et al. included 124 VATS pneumonectomy to 235 open operations. Outcomes mirrored prior studies with a 19% conversion rate, and persistent differences are found only on the extent of lymph node dissection leaning in favor of the VATS approach (40) (Table 5). Data on robotic resections are sparse. Only six have been described with various configurations. Outcomes include two conversions and one complication/mortality from pulmonary artery bleeding (41) (Table 6).

**Table 5:** Outcomes of video-assisted thoracoscopic pneumonectomy.

**Trial**	**Study type**	**Date**	***n***	**Findings**
Battoo et al. (38)	Single-center cohort	2002–2012	107 67 VATS 40 Open	289 VATS vs. 225 min open OR median OR time 7.5 VATS vs. 5% open Op mortality, not significant No difference in complication rate, locoregional recurrence, or LOS 17 conversions to open 54 VATS vs. 19% open pain free at 1 year
Liu et al. (39)	Single-center cohort	2013–2016 VATS 2010–2013 open	96 (2:1 matched) 32 VATS 64 open	0 VATS vs. 0% open Op mortality No difference in complications, transfusion, EBL, drainage time, LOS 187 VATS vs. 146 OR time 2.1 VATS vs. 2.6 open, mean pain score
Yang et al. (40)	Multi-center cohort	2000–2016	359 124 VATS 235 open	7 VATS vs. 8% open, Op mortality 28 VATS vs. 28% open, complication rate 47 VATS vs. 33% open, not significant, 5-year survival 19% conversion to open

**Table 6:** Case series of robotic pneumonectomy.

**Trial**	**Study type**	***n***	**Findings**
Giulianotti et al. (42)	Case series	3	2 converted, 1 death from PA injury and bleed
Spaggiari et al. (43)	Case series	2	No morbidity or complication
Rodriguez et al. (44)	Case report	1	No morbidity or complication

### Robotic Technique for Pneumonectomy

Robotic pneumonectomy raises a number of technical concerns. Exposure of the main pulmonary artery often requires division of the superior pulmonary vein from an anterior approach. With large hilar tumors, proximal extent of dissection may require intra-pericardial dissection. Early division of the pulmonary veins can engorge the specimen and make extraction difficult, require extra maneuvers including piecemeal extraction, and threaten tumor seeding. Left pneumonectomy is hampered by the aorta and risk of a long bronchial stump. Full dissection of the aortopulmonary window lymph nodal tissue can aid in retraction and allow a more proximal staple line. However, we have now performed eight in the past 2 years with success (video of our technique available at https://www.youtube.com/watch?v=P1mrftUCBCg).

In our experience, an intra-pericardial approach is often if not always needed, since a sleeve is not possible, and can facilitate division of pulmonary vasculature. After most of the dissection around the pulmonary vascular is performed, and gross or multi-station N2 disease is ruled out, we generally divide the inferior vein first. Next, the superior pulmonary vein and then the main pulmonary artery are divided. When performing left pneumonectomy, complete dissection of the subcarinal and AP window nodes allows for a bronchial division at the carina with a black handheld staple load, which is performed last. If possible, we routinely divide the specimen prior to extraction to extract two smaller specimens, rather than one large one to limit incision side or use of rib spreaders. If the specimen is divided, we routinely divide along the fissure.

## Discussion

As discussed in detail above, lung cancer remains a growing source of morbidity and mortality in this country, demonstrated by its position as the leading cause of cancer death among Americans. With innovation in medical therapy, we are able to extend life and avoid morbidity in more advanced-stage NSCLC than previously. Surgical resection has always been an important therapy for NSCLC, and its role in advanced disease post-induction is an evolving endeavor that will need close attention in the coming years. With the advent of the robotic platform, these operations, as well as more extended resections, such as pneumonectomy and sleeve lobectomy, may be able to be performed in a way to reap the benefits of the more localized resections completed in a minimally invasive manner. With the data reviewed above, we were able to demonstrate that an adequate resection is indeed achievable. R0 resection rates were similar in the largest post-induction study we reviewed, and an MIS sleeve lobectomy 20-person study revealed a 100% R0 resection rate. Moreover, although only a small handful of MIS studies exist, short- and long-term survival rates appeared to be statistically similar between an MIS and open approach for these extended resections. Nonetheless, these few studies also seem to have a similar trend of low conversion-to-thoracotomy rate. This was reported to be as low as 4.5%, but tended to be on average around 20% between the reviewed studies. This most often was due to bleeding and/or heavily calcified and fibrotic hilar nodes creating a more dangerous dissection.

Overall, we believe that this review of the data and our institution's experience prove that these extended resections are feasible using a robotic platform. The most important aspect is preparation on part of the surgeon, as well as knowledge of what to expect intra-operatively. Our group has a focused strategy on these essential steps: (1) obtaining hilar control prior to proceeding with the areas of most complicated dissection, especially in the post-induction cases; (2) a thorough LN dissection to create a definitive picture of airway anatomy, as well as lining up the airway appropriately to suture; and (3) intra-pericardial hilar control for the MIS pneumonectomy. We believe that if the surgical team is able to follow these key steps, while always being prepared to open for thoracotomy (i.e., blood products on hold, thoracotomy tray in the room, sponges in the chest for pressure, senior partners nearby), these resections can be performed in a safe and efficacious manner to best treat this subset of patients.

## Author Contributions

JS and JJ: writing, editing, and research. SC, TG, and RC: writing and editing. All authors contributed to the article and approved the submitted version.

## Conflict of Interest

RC discloses past relationships with AstraZeneca, Bard Davol, Bovie Medical Corporation, C-SATS, ConMed, Covidien/Medtronic, Ethicon, Fruit Street Health, Google/Verb Surgical, Intuitive Surgical, KCI/Acelity, Myriad Genetics, Neomend, Pinnacle Biologics, ROLO-7, Tego, and TransEnterix. The remaining authors declare that the research was conducted in the absence of any commercial or financial relationships that could be construed as a potential conflict of interest.
